# Simple and efficient estimation of photovoltaic cells and modules parameters using approximation and correction technique

**DOI:** 10.1371/journal.pone.0216201

**Published:** 2019-05-02

**Authors:** Fahmi F. Muhammad, Ali W. Karim Sangawi, Suhairul Hashim, S. K. Ghoshal, Isam K. Abdullah, Shilan S. Hameed

**Affiliations:** 1 Department of Physics, Faculty of Science, Universiti Teknologi Malaysia (UTM), Skudai, Johor, Malaysia; 2 Nanotechnology Research Center, Cihan University-Erbil, Erbil, Kurdistan Region, Iraq; 3 College of Basis Education, Charmo University, Chamchamal, Kurdistan Region, Iraq; 4 Centre for Sustainable Nanomaterials (CSNano), Ibnu Sina Institute for Scientific and Industrial Research (ISI-SIR), Universiti Teknologi Malaysia (UTM), Skudai, Johor, Malaysia; 5 Department of Physics, College of Science, Salahaddin University, Erbil, Kurdistan Region, Iraq; 6 Directorate of Information Technology, Koya University, Koya, Kurdistan Region, Iraq; Politecnico di Milano, ITALY

## Abstract

The behavior of solar cells and modules under various operational conditions can be determined effectively when their intrinsic parameters are accurately estimated and used to simulate the current-voltage (*I-V*) characteristics. This work proposed a new computational approach based on approximation and correction technique (ACT) for simple and efficient extraction of solar cells and modules parameters from the single-diode model. In this technique, an approximated value of series resistance (*R*_*s*_) was first derived and used to determine the initial value of parallel resistance (*R*_*p*_). Later, the final corrected values of *R*_*s*_ and *R*_*p*_ were obtained by resubstituting their approximated values in a five-loop iteration using the manipulated equations. For rapid evaluation and validation of the proposed technique, a software application was also created using MATLAB program. The correctness and robustness of the proposed technique was validated on five types of solar cells and modules operated at varied temperatures and irradiances. The lowest RMSE value was achieved for RTC France (7.78937E-4) and PVM 752 GaAs (2.10497E-4) solar cell. The legitimacy of ACT extracted parameters was established using a simple yet competitive implementation approach wherein the performance of the developed technique was compared with several state-of-the-art methods recently reported in the literature.

## Introduction

The utilization of photovoltaic (PV) technology to convert sunlight energy into electricity via various solar cell devices has increasingly been demanded in public sectors, industries, and space program [1–4]. This is largely due to easy installation and low maintenance cost of photovoltaic electricity compared to other electricity sources. PV modules are made of cells connected in series and parallel to provide the desired current and voltage. However, the performance of these devices can be affected by the change in temperature, sunlight intensity and aging [5–7]. Hence, it is important to study and analyze comprehensively the output characteristics of such cells and modules under various conditions using appropriate model [8, 9]. This in turn can help the users to take effective strategies for delivering safely the maximum power to the electrical appliances/loads.

**Fig 1:**
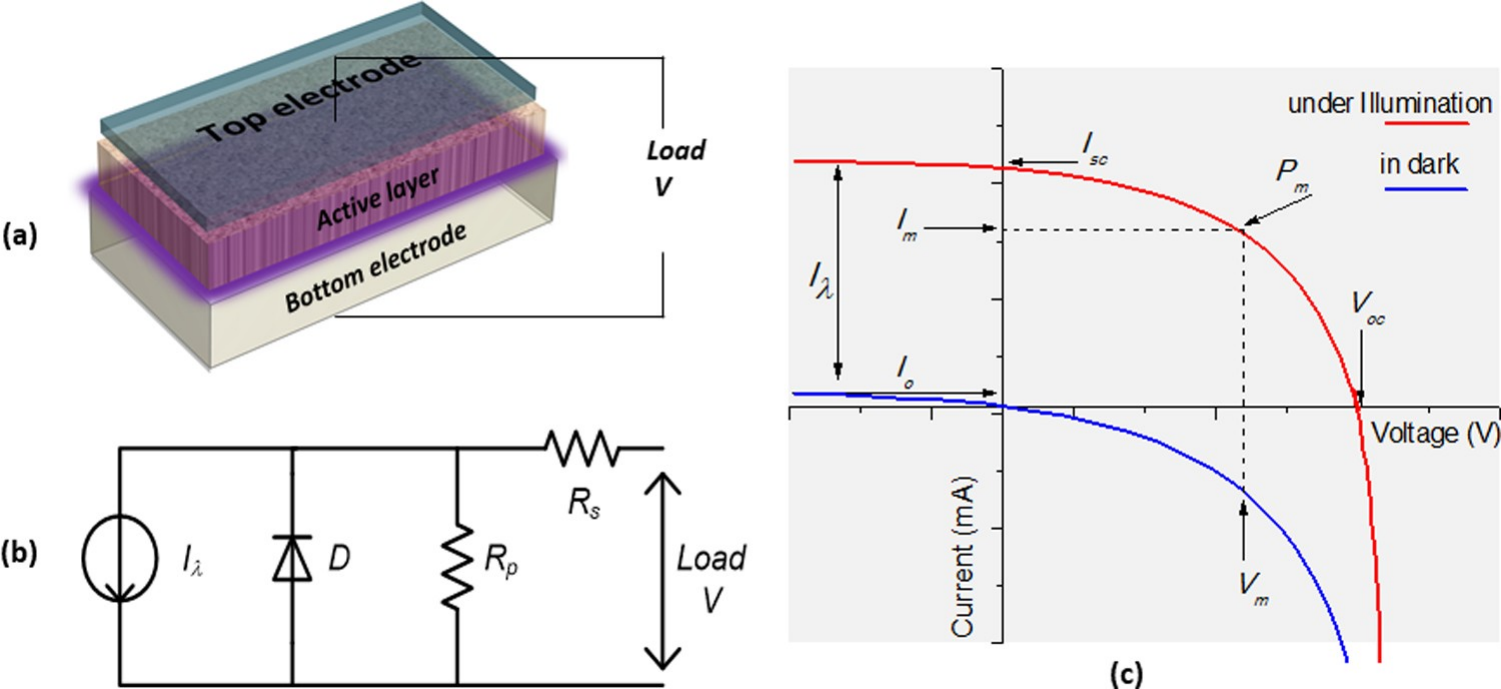
Prototype structure of solar cells (a), the equivalent circuit used to simulate *I-V* curve of solar cells based on single-diode model (b) and the standard *I-V* characteristic of solar cells (c).

Single-diode and double-diode models have widely been used to simulate the PV characteristics [8, 10, 11]. In order to make a successful performance prediction, it is essential to simulate accurately the behavior of real-world PV devices to represent their current-voltage (*I-V*) characteristics. The single-diode model is comprised of an ideal diode connected in parallel with a constant current source and a shunt resistance driven to an external load through a series resistance (see Fig 1B). Such single-diode model can accurately present the conventional solar cells behavior [12, 13], wherein the unknown model parameters correspond to the PV cells or modules parameters. Estimation of these solar cells or modules parameters based on the experimental *I-V* data is very significant for performance assessment, simulation, design, and quality control [14].

The implicit nature of single-diode model equation makes the simple and accurate determination of these parameters somewhat challenging. Therefore, different approaches have been proposed to extract the solar cells and modules parameters including analytical techniques, computational methods and a combination of both. Analytical methods are less accurate either due to the utilization of fewer points of the practical *I-V* data or the negligence of some terms of the applied equations during the parameters extraction process [15–22]. Generally, highly accurate extraction of PV cell parameters is performed via numerical fitting of the practical *I-V* data using Newton-Raphson method [23], Levenberg–Marquardt algorithm [24], polynomial curve fitting technique and Lambert-W function [25] or conductivity method [26]. Nonetheless, these techniques can be easily trapped into local optimum because of their sensitivity to the chosen values of initial parameters. To surmount such limitations, various approaches have been developed for accurate and efficient estimation of PV device parameters.

Lately, stochastic computational optimization based on evolutionary and heuristic algorithms became prospective owing to their capacity of handling nonlinear equations and global search pattern. For instance, differential evolution (DE) technique [27, 28], bird mating optimizer (BMO) [29–31], genetic algorithm (GA) [32, 33], artificial bee colony (ABC) [34] and particle swarm optimization (PSO) [35, 36] have successfully been utilized to estimate the PV cells and modules parameters. Basically, these algorithms work on a random selection of the intended parameters simultaneously within a large solution space, ultimately leading to high computational cost and reduced stability. On top, the solution may easily get stuck into the local optimum when the dimension of the objective function is large [37]. Thus, dedicated efforts have been made to surmount the limitations of stochastic computational techniques through the establishment of alternative algorithms and internal parametric modifications. Examples of these approaches are PSO with binary constraints [38], guaranteed convergence PSO [39], improved chaotic whale optimization algorithm [40], improved shuffled complex evolution algorithm [37] and hybrid firefly algorithm with pattern search algorithm (HFAPS) [41]. Additionally, with the aim of combining the simplicity of analytical methods and efficiency of computational techniques various hybrid approaches have been introduced to estimate the PV cells and modules parameters [18, 21, 42, 43]. Laudani *et al*. reported an efficient parameter extraction for the single-diode model from experimental *I-V* data using a reduced form [44], where the equations was numerically solved by non-linear least square technique. Interestingly, this approach provided low computational cost and excellent convergence. However, the method is limited when it comes to quantify the PV parameters *I*_*sc*_, *V*_*oc*_, *V*_*m*_ and *I*_*m*_ for their use in extracting the solar cells and modules parameters. It has been realized that in addition to the experimental *I-V* data, a separate procedure needs to be performed to find the PV parameters or they have to be obtained either from datasheet information or from literature [45].

The performance and accuracy of the existing techniques for the estimation of the single-diode parameters is known to be limited due to their implementation complexity and reproducibility. Besides, these computational approaches can affect the simulated *I-V* curve that deviates from the experimental one which is majorly due to the high sensitivity of *I-V* characteristic on the ideality factor and series resistance [8]. Thus, special strategies must be taken to the initial estimation of the ideality factor and series resistance while implementing the derived equations. To resolve such problems, present work proposes a new computational approach based on approximation and correction technique (ACT) for simple and efficient determination of the solar cells and modules parameters. MATLAB program was used for rapid evaluation and validation of the proposed technique. This technique was implemented through the use of few viable equations and the competitive accuracy of the simulated *I-V* compared to the experimental ones at different environmental conditions. The developed method was shown to require a set of *I-V* data without manual feeding of PV parameters (*I*_*sc*_, *V*_*oc*_, *V*_*m*_ and *I*_*m*_) and used the extrapolation fit to estimate such parameters prior to the initialization. The outperforming attributes of newly proposed technique were established.

## Methodology

### Mathematical manipulations

PV cells and modules that are modeled by an equivalent electrical circuit can simulate their current-voltage (*I-V*) characteristics. Fig 1A shows a prototype structure of solar cells, while Fig 1B and 1C present the equivalent circuit along with its standard *I-V* characteristic curves in dark and under illumination, respectively. The light activated current source (*I*_*λ*_) depicts the amount of current generated in the cell when it is exposed to photon energy.

The application of a load across the right side of the circuit (Fig 1B) gives rise to a potential difference, which ultimately acts upon reducing the total amount of current passing through it in a reverse biased scheme. As such, the summation of instantaneous current in the load determines the final characteristic current of the device. Therefore, the net current of the equivalent circuit so called the output generated current of solar cells can be represented by Eq 1:

$$I=I_{\lambda }-I_{o}\left[\exp\left(\frac{V+IR_{s}}{nV_{t}}\right)-1\right]-\frac{\left(V+IR_{s}\right)}{R_{p}}$$ (1)

where *n* is the ideality factor of the solar cell signifying the charge transport efficiency of the device, $I_{o}$ is the saturation (generation) current of the diode under dark conditions, *V*_*t*_ is the thermal voltage represented by $k_{B}T/q,k_{B}$ is the Boltzmann’s constant, *T* is the temperature in Kelvin, *q* is the elementary charge and *R*_*s*_ and *R*_*p*_ are the respective series and parallel resistance. For a PV module, $\left(V+IR_{s}\right)$ becomes $\left(V/N_{s}+IR_{s}\right)$ since PV module is composed of *N*_*s*_ series connected cells. Consequently, the denominator of the exponent part in the equation is involved with *N*_*s*_. Thus, during the numerical iterations, (*N*_*s*_ × *n*) should be considered instead of *n*.

Using three boundary conditions obtained from the *I-V* curve (Fig 1C) such as the open circuit voltage (*V*_*oc*_), the short circuit current (*I*_*sc*_) and the maximum power (*P*_*m*_), Eq 1 can be transformed as:

$$0=I_{\lambda }+I_{o}-I_{o}\exp\left(\frac{V_{oc}}{nV_{t}}\right)-\frac{V_{oc}}{R_{p}}$$ (2)

$$I_{sc}=I_{\lambda }+I_{o}-I_{o}\exp\left(\frac{R_{s}I_{sc}}{nV_{t}}\right)-\frac{R_{s}I_{sc}}{R_{p}}$$ (3)

$$I_{m}=I_{\lambda }+I_{o}-I_{o}\exp\left(\frac{R_{s}I_{m}+V_{m}}{nV_{t}}\right)-\frac{R_{s}I_{m}}{R_{p}}-\frac{V_{m}}{R_{p}}$$ (4)

Combining Eqs 2 from 3 one gets the underneath expression saturation current (*I*_*o*_):

$$I_{o}=\frac{I_{sc}-\frac{V_{oc}}{R_{p}}+\frac{R_{s}}{R_{p}}I_{sc}}{\exp\left(\frac{V_{oc}}{nV_{t}}\right)-\exp\left(\frac{R_{s}I_{sc}}{nV_{t}}\right)}$$ (5)

The second exponential term in the denominator due to its smallness for almost all the types of solar cells [13, 18, 46, 47] can be approximated without loss of generality and transforms the relation to:

$$I_{o}=\frac{I_{sc}-\frac{V_{oc}}{R_{p}}+\frac{R_{s}}{R_{p}}I_{sc}}{\exp\left(\frac{V_{oc}}{nV_{t}}\right)}$$ (6)

Under the same approximation Eq 3 yields:

$$I_{\lambda }=I_{sc}-I_{o}+\frac{R_{s}I_{sc}}{R_{p}}$$ (7)

Substituting Eqs 6 and 7 into Eq 4 and solving for *R*_*s*_ one gets:

$$R_{s}=\frac{\left(nV_{t}\right)\ln\left(\frac{I_{sc}-I_{m}-(V_{m}/R_{p})-(R_{s}I_{m}/R_{p})+(R_{s}I_{sc}/R_{p})}{I_{sc}-(V_{oc}/R_{p})+(R_{s}I_{sc}/R_{p})}\right)-V_{m}+V_{oc}}{I_{m}}$$ (8)

A boundary condition at the maximum power point of the *I-V* curve can be applied to derive the explicit form of *R*_*p*_ wherein the internal impedance (*Z*_*in*_) of the equivalent circuit is taken equal to the impedance of the external load (*Z*_*out*_). From the theorem of maximum power transfer to the external load one achieves,

$$Z_{in}=Z_{out}=\frac{V_{m}}{I_{m}}$$ (9)

Based on the equivalent circuit, the impedance function takes the form:

$$\frac{R_{p}r_{d}}{R_{p}+r_{d}}+R_{s}=\frac{V_{m}}{I_{m}}$$ (10)

where *r*_*d*_ is the dynamic resistance of the diode at maximum power point (*P*_*max*_) which is determined from the first derivative of the diode voltage with respect to its current given by:

$$r_{d}={\frac{dV_{D}}{dI_{D}}|}_{P_{m}}=\frac{nV_{t}}{I_{o}exp\left(\frac{R_{s}I_{m}+V_{m}}{nV_{t}}\right)}$$ (11)

Solving Eqs 11 and 10 one gets:

$$I_{o}\exp\left(\frac{R_{s}I_{m}+V_{m}}{nV_{t}}\right)=\frac{nV_{t}(I_{m}-\frac{V_{m}}{R_{p}}+\frac{R_{s}I_{m}}{R_{p}})}{V_{m}-R_{s}I_{m}}$$ (12)

Manipulating Eqs 2, 4 and 6 one achieves:

$$I_{o}\exp\left(\frac{R_{s}I_{m}+V_{m}}{nV_{t}}\right)=I_{sc}-I_{m}+\frac{R_{s}}{R_{p}}\left(I_{sc}-I_{m}\right)-\frac{V_{m}}{R_{p}}$$ (13)

Using Eqs 12 and 13, the explicit form of *R*_*p*_ can be obtained as:

$$R_{p}=\frac{V_{m}^{2}+R_{s}^{2}\left(I_{sc}I_{m}-I_{m}^{2}\right)+R_{s}\left(nV_{t}I_{m}-I_{sc}V_{m}\right)-nV_{t}V_{m}}{R_{s}\left(I_{m}^{2}-I_{sc}I_{m}\right)+V_{m}\left(I_{sc}-I_{m}\right)-nV_{t}I_{m}}$$ (14)

### Method formulation

The main concept of ACT approach is emerged from the fact that the value of every dependent variable *y* = *f(x)* in an implicit function can be approximated by neglecting initially the part of the equation that contains *y*. Let us assume two simultaneous implicit equations, where the first one is y=a+ln⁡(b+cyx) and the second one is $x=dy+ey^{2}$, where *a*, *b*, *c*, *d* and *e* are some defined constants. Now, to find the approximate value of *y* in the first implicit equation, the term *cy/x* is initially neglected. This approximate value of *y* can be utilized in the second equation to guess the approximate value of *x*. Then, these approximate values of *x* and *y* can be resubstituted into the neglected part (*cy/x*) at the right side of the implicit equation to determine the new value of *y* at the left side. In this way, the resubstituting process within few iterative loops allows to exact the values of *y* and *x*. Driven by this proposed approach, the rational terms including *R*_*p*_ in Eq 8 are initially neglected to determine the approximate values of initial *R*_*s*_ (*R*_*s_i*_) via:

$$R_{s\_i}=\frac{\left(nV_{t}\right)\ln\left(1-\frac{I_{m}}{I_{sc}}\right)-V_{m}+V_{oc}}{I_{m}}$$ (15)

**Fig 2:**
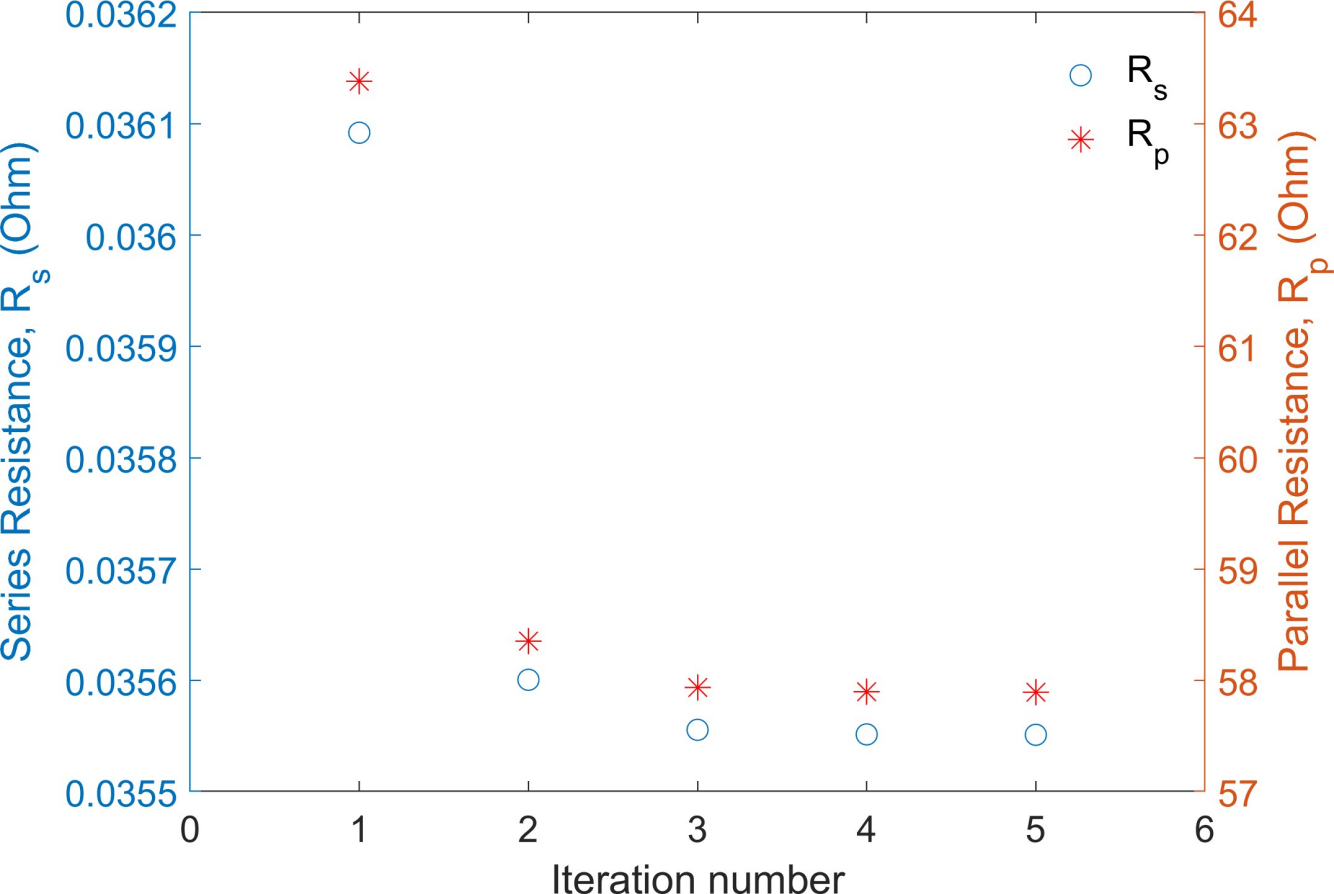
The change in values of *R*_*s*_ and *R*_*p*_ within five loop iterations, the final fixed values are used in ACT approach for the parameters extraction.

Value of *R*_*s_i*_ was utilized to estimate the preliminary value of *R*_*p*_ using Eq 14. Later on, the final corrected values of *R*_*s*_ and *R*_*p*_ were obtained through resubstituting their approximated values in a five-loop iteration procedure using Eqs 8 and 14, respectively. It was found that five iterations were sufficient to achieve a fixed/corrected value of *R*_*s*_ and *R*_*p*_ (Fig 2). The value of *R*_*s*_ being always smaller than *R*_*p*_ by several orders of magnitude allows one to resubstitute *R*_*s*_ into Eq 14 for modifying *R*_*p*_.

Eq 5 was used to determine the saturation current (*I*_*o*_) and Eq 2 was exploited to extract the photocurrent given by:

$$I_{\lambda }=I_{o}\left[\exp\left(\frac{V_{oc}}{nV_{t}}\right)-1\right]+\frac{V_{oc}}{R_{p}}$$ (16)

### Method implementation and validation

The ACT approach was implemented using MATLAB code (runtime R2017b) on a PC (core i7 and 8 GB RAM) involving the following steps:

1. The measured *I-V* data was imported in csv format and a new dataset of power-voltage (*P-V*) is generated from. Then, exponential, Gaussian and polynomial interpolation techniques were utilized around the respective points *I* = 0, *V* = 0 and *P* = *P*_*max*_ to determine the corresponding photovoltaic parameters *I*_*sc*_, *V*_*oc*_, *V*_*m*_ and *I*_*m*_.
2. Number of series cells and operating temperature of the device was fed as input data.
3. A set of ideality factor (*n*) was initialized, starting from 0.5 to 6 with an increment of 0.1.
4. At every point of *n*, Eq 15 was used to determine the approximate value of *R*_*s*_, while the final corrected values of *R*_*p*_ and *R*_*s*_ were calculated via the proposed ACT method using Eqs 14 and 8.
5. The values of *I*_*o*_ and *I*_*λ*_ were also determined from Eqs 5 and 16, respectively.
6. At every value of *n*, a set of simulated *I-V* data was generated based on the estimated parameters using a non-linear zero finding (*fzero*).
7. The set of PV cell/module parameters that provided the best fitting of the simulated *I-V* data to that of the experimental ones (lowest RMSE) was chosen as the optimum solution.
8. Similarly, a new set of *n* was initialized around the best previous chosen *n* (at which the optimum parameters were extracted) with the increment of 0.01, while steps 4 to 7 were repeated to improve the accuracy and efficiency of the method.
9. Step 8 was repeated for five sequential iterations, where the value of *n* with five decimal orders was obtained.

**Fig 3:**
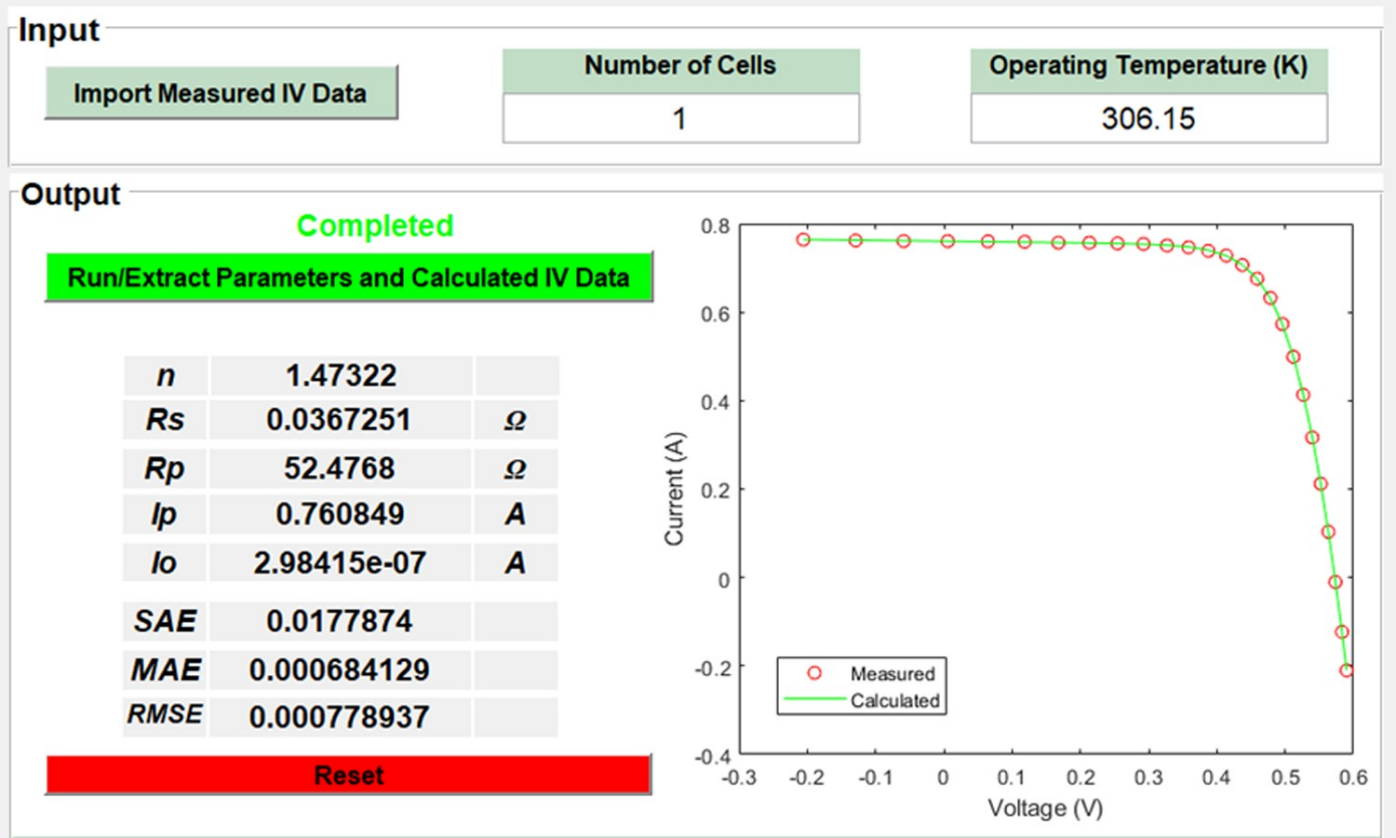
The workspace of the established software application, which is used to extract the PV cells and modules parameters based on ACT.

The coding was deployed in a stand-alone software application for simple and fast implementation of the above steps. This allowed the efficient extraction of the PV cells and modules parameters. Fig 3 shows the workspace of this application, where the given inputs to the app were measured *I-V* data in csv format, number of series cells and operating temperature. The application provided a fast and efficient extraction of the parameters that could calculate the *I-V* data within few seconds. This application was made freely available to users as a supporting file (S1 File). The app can be executed using the free available software (MATLAB runtime R2017b) and without MATLAB installation in the computer (https://www.mathworks.com/products/compiler/matlab-runtime.html).

The performance proposed method was validated using five PV cells and modules (composed of different series cells) operated under different temperatures and solar irradiations. These include France solar cell operated at 33°C and irradiation of 1000 W/m^2^ (measured *I-V* data was provided in S1 Dataset), PVM 752 GaAs thin film cell operated at 25°C and irradiation of 1000 W/m^2^ (measured *I-V* data was provided in S2 Dataset), Photowatt-PWP201 module comprising 36 polycrystalline silicon cells in series operated at 45°C and 1000 W/m^2^ (measured *I-V* data was provided in S3 Dataset), Leibold Solar Module (STE 20/100) comprising 20 series cells operated at 24°C under outdoor sunlight intensity of 360 W/m^2^ (measured *I-V* data was provided in S4 Dataset) and a four series cells-based polycrystalline PV module (STE 4/100) from Leybold GmbH operated at 22°C under indoor light intensity of 900 W/m^2^ (measured *I-V* data was provided in S4 Dataset).

The accuracy of the proposed method was assessed correspondingly in terms of summation of absolute error (SAE), mean of absolute error (MAE) and root mean squared error (RMSE) given by the relations:

$$SAE=\overset{k}{\underset{i=1}{\sum }}\left|\left(I_{i}^{meas}-I_{i}^{cal}\right)\right|$$ (17)

$$MAE=\frac{1}{k}\overset{k}{\underset{i=1}{\sum }}\left|\left(I_{i}^{meas}-I_{i}^{cal}\right)\right|$$ (18)

$$RMSE=\sqrt{\frac{1}{k}\overset{k}{\underset{i=1}{\sum }}{\left(I_{i}^{meas}-I_{i}^{cal}\right)}^{2}}$$ (19)

where *k* is the number of elements in the measured *I* dataset, $I_{i}^{meas}$ and $I_{i}^{cal}$ are the *i*^*th*^ measured current and calculated current, respectively.

## Results and discussion

The performance of proposed ACT was validated and assessed using five types of PV devices (two PV cells and three PV modules) at different device temperatures and solar irradiations. Consequently, the extracted PV cells and modules parameters were recorded and utilized to generate simulated *I-V* data for each type of the device. The correctness and robustness of ATC was tested by comparing with the existing art of the techniques reported in the literature.

### Parameters of PV cells

Commercially available R.T.C. France solar cell (57 mm of diameter operated at 33°C) was tested for the parameters extraction and validation of the proposed method [23]. The measured *I-V* data of the cell was given in csv format as the S1 Dataset. Table 1 depicts the simulated currents and the ACT derived absolute error between the experimental and simulated currents. The calculated currents agreed very well with the measured ones.

**Table 1:** Calculated/simulated results for single-diode model of R.T.C. France solar cell obtained using ACT.

Voltage (V)	Measured Current (A)	Calculated Current (A)	Absolute Error (AE)
-0.2057	0.7640	0.7642	0.00023
-0.1291	0.7620	0.7628	0.00078
-0.0588	0.7605	0.7614	0.00094
0.0057	0.7605	0.7602	0.00029
0.0646	0.7600	0.7591	0.00092
0.1185	0.7590	0.7580	0.00095
0.1678	0.7580	0.7571	0.00092
0.2132	0.7570	0.7561	0.00089
0.2545	0.7555	0.7550	0.00045
0.2924	0.7540	0.7536	0.00038
0.3269	0.7505	0.7514	0.00087
0.3585	0.7465	0.7474	0.00087
0.3873	0.7385	0.7402	0.00168
0.4137	0.7280	0.7276	0.00044
0.4373	0.7065	0.7072	0.00071
0.4590	0.6755	0.6756	0.00011
0.4784	0.6320	0.6312	0.00080
0.4960	0.5730	0.5723	0.00066
0.5119	0.4990	0.4996	0.00065
0.5265	0.4130	0.4135	0.00052
0.5398	0.3165	0.3171	0.00063
0.5521	0.2120	0.2119	5.8E-05
0.5633	0.1035	0.1026	0.00095
0.5736	-0.0100	-0.0090	0.00064
0.5833	-0.1230	-0.1240	0.00135
0.5900	-0.2100	-0.2090	0.00097

Table 2 compares the extracted parameters of R.T.C France solar cell obtained using the ACT with other reported methods in literature. Vales of SAE, MAE and RMSE indicated a highly competitive performance of the ACT and its reliance to determine accurately the PV cells and modules parameters. It is worth mentioning that the developed ACT outperformed several recently reported stochastic and deterministic optimization algorithms such as PGJAYA, ILCOA, HFAPS, ISCE and IJAYA in terms of accuracy, simplicity and computational cost (rapidity). The proposed ACT was able to extract the devices parameters in few seconds, while almost all the stochastic algorithms require several minutes. The reduced form (RF) approach [44] was also seen to provide low computational cost and excellent convergence. However, this method is limited when it comes to quantify the PV parameters *I*_*sc*_, *V*_*oc*_, *V*_*m*_ and *I*_*m*_ before their utilization in the process of extracting the solar cells and modules parameters. Alternatively, these parameters need to be obtained manually either from datasheet information or from literature. Present ACT can automatically estimate all the PV parameters during the first *I-V* screening, while extracting the devices parameters accurately and efficiently.

**Table 2:** Comparison of different parameters extraction methods for single-diode model of R.T.C. France solar cell.

Methods	*n*	*R*_*s*_ (Ω)	*R*_*p*_ (Ω)	*I*_*λ*_(A)	*I*_*o*_ (μA)	SAE	MAE	RMSE
ACT (proposed)	1.47322	0.0367251	52.4768	0.760849	0.298415	17.7874E-3	6.84129E-4	7.78937E-4
RF [44]	1.4769641	0.03655304	52.859056	0.7607884	0.3102482	NA	**6.7810E-4**	**7.7301E-4**
PGJAYA [48]	1.4812	0.0364	53.7185	0.7608	0.3230	NA	NA	9.8602E-4
ILCOA [49]	1.481108	0.036377	53.718679	0.760775	0.323021	NA	NA	9.86021E-4
HFAPS [41]	1.48106	0.0363819	53.6784	0.760777	0.322622	NA	NA	9.8602E-04
IJAYA [50]	1.4811	0.0364	53.7595	0.7608	0.3228	NA	NA	9.8603E-4
ISCE [37]	1.48118360	0.03637709	53.71852771	0.76077553	0.32302083	17.70412E-3	NA	9.860219E-4
EHA-NMS [51]	1.48118359	0.03637709	53.71852139	0.76077553	0.32302080	17.70412E-3	NA	9.860219E-4
R_cr_-IJADE [27]	1.48118360	0.03637709	53.71852500	0.76077553	0.32302080	**17.70357E-3**	NA	9.86022E-4
PCE [52]	1.481074	0.036377	53.718525	0.760776	0.323021	NA	NA	9.86022E-4
ABC [34]	1.4817	0.0364	53.6433	0.7608	0.3251	NA	8.3034E-4	9.8620E-4
GOTLBO [53]	1.483820	0.036265	54.115426	0.760780	0.331552	NA	NA	9.87442E-4
CSO [54]	1.48118	0.03638	53.7185	0.76078	0.3230	NA	6.7968E-4	9.8602E-4
STLBO [55]	1.48114	0.03638	53.7187	0.76078	0.32302	NA	8.29E-4	9.8602E-4
ABC-DE [56]	1.47986	0.03637	53.7185	0.76077	0.32302	NA	NA	9.8602E-4
BMO [30]	1.48173	0.03636	53.8716	0.76077	0.32479	NA	4.621E-3	9.8608E-4
NM-MPSO [57]	1.48120	0.03638	53.7222	0.76078	0.32306	NA	4.598E-3	9.8602E-4
MABC [58]	1.481385	0.036389	53.39999	0.760779	0.321323	NA	8.3118E-4	9.8610E-4
GGHS [59]	1.48217	0.03631	53.0647	0.76092	0.32620	NA	4.6E-3	9.9097E-4
ABSO [60]	1.47583	0.03659	52.2903	0.76080	0.30623	NA	NA	9.9124E-4
IGHS [59]	1.48740	0.03613	53.2845	0.76077	0.34351	NA	NA	9.9306E-4
CPSO [61]	1.5033	0.0354	59.012	0.7607	0.4000	NA	16.8E-2	1.3900E-3
CWOA [40]	1.4812	0.03636	53.7987	0.76077	0.3239	NA	8.28E-4	9.8604E-4

The PVM 752 GaAs thin film cell (operated at 25°C and irradiation of 1000 W/m^2^) was tested to extract its parameters [62]. Yet again, the measured *I-V* data of this cell was given in csv format as the S2 Dataset. The calculated currents were observed to be in very good agreement with the measured ones (Table 3) when compared with those reported in the literature. Besides, ACT demonstrated excellent performance to determine the PV parameters of the cell (Table 4).

**Table 3:** Calculated/simulated results for single-diode model of PVM 752 GaAs thin film cell achieved using ACT.

Voltage (V)	Measured Current (A)	Calculated Current (A)	Absolute Error (AE)
-0.1659	0.1001	0.1001	3.7E-05
-0.1281	0.1000	0.1001	8.2E-05
-0.0888	0.0999	0.1000	0.00012
-0.0490	0.0999	0.1000	6.7E-05
-0.0102	0.0999	0.0999	9.9E-06
0.0275	0.0998	0.0999	5.5E-05
0.0695	0.0999	0.0998	0.00011
0.1061	0.0998	0.0997	6E-05
0.1460	0.0998	0.0997	0.00012
0.1828	0.0997	0.0996	7.2E-05
0.2230	0.0997	0.0996	0.00013
0.2600	0.0996	0.0995	8.5E-05
0.3001	0.0997	0.0995	0.00024
0.3406	0.0996	0.0994	0.0002
0.3789	0.0995	0.0993	0.00016
0.4168	0.0994	0.0993	0.00011
0.4583	0.0994	0.0992	0.00018
0.4949	0.0993	0.0992	0.00013
0.5370	0.0993	0.0991	0.0002
0.5753	0.0992	0.0990	0.00018
0.6123	0.0990	0.0989	7E-05
0.6546	0.0988	0.0988	4.3E-05
0.6918	0.0983	0.0985	0.00017
0.7318	0.0977	0.0978	0.00011
0.7702	0.0963	0.0964	1E-04
0.8053	0.0937	0.0937	2E-06
0.8329	0.0900	0.0898	0.00016
0.8550	0.0855	0.0851	0.00045
0.8738	0.0799	0.0794	0.00046
0.8887	0.0743	0.0738	0.00049
0.9016	0.0683	0.0680	0.00028
0.9141	0.0618	0.0616	0.00024
0.9248	0.0555	0.0554	0.00014
0.9344	0.0493	0.0493	1.3E-05
0.9445	0.0422	0.0424	0.00018
0.9533	0.0357	0.0359	0.00024
0.9618	0.0291	0.0294	0.00026
0.9702	0.0222	0.0225	0.00033
0.9778	0.0157	0.0161	0.00038
0.9852	0.0092	0.0096	0.00037
0.9926	0.0026	0.0028	0.00024
0.9999	-0.0040	-0.0040	1.1E-05
1.0046	-0.0085	-0.0090	2.4E-05
1.0089	-0.0124	-0.0130	0.00032

**Table 4:** Comparison of different parameter extraction methods with the proposed ACT tested for single-diode model of PVM 752 GaAs thin film cell.

Methods	*n*	*R*_*s*_ (Ω)	*R*_*p*_ (Ω)	*I*_*λ*_(A)	*I*_*o*_ (pA)	SAE	MAE	RMSE
ACT (proposed)	1.73411	0.616566	684.519	0.099985	19.4231	**7.42586E-3**	**1.6877E-4**	**2.10497E-4**
ELPSO [62]	1.768590	0.159052	14.429507	0.115016	0	NA	NA	2.5400E-2
CPSO [61]	1.617093	0.346578	14.241982	0.116530	0	NA	NA	2.5400E-2
BSA [62]	1.858574	0.5	100	0.103903	84.90	NA	NA	2.1469E-3
ABC [62]	1.774159	0.5	100	0.103312	32.00	NA	NA	2.0412E-3

Table 4 summarizes the parameters of PVM 752 GaAs thin film cell extracted by ACT when compared with other works. The values of SAE, MAE and RMSE clearly revealed the high competitive accuracy of ACT and its strong trustworthiness for the accurate evaluation of PV cells and modules parameters.

### Parameters of PV modules

In addition to PV cells, three different solar modules were examined to extract their parameters and to validate the proposed ACT. Photowatt-PWP201 (comprised of 36 polycrystalline silicon cells in series and operated at 45°C and 1000 W/m^2^) [23] was used wherein the measured *I-V* data is given in csv format as the S3 Dataset. Table 5 enlists the calculated *I-V* results using ACT and Table 6 summarizes the extracted parameters.

**Table 5:** Calculated/simulated results for single-diode model of Photowatt-PWP201 module tested using ACT.

Voltage (V)	Measured Current (A)	Calculated Current (A)	Absolute Error (AE)
-1.9426	1.0345	1.0332	0.00127
0.1248	1.0315	1.0305	0.00098
1.8093	1.0300	1.0283	0.00171
3.3511	1.0260	1.0262	0.00020
4.7622	1.0220	1.0242	0.00216
6.0538	1.0180	1.0220	0.00400
7.2364	1.0155	1.0193	0.00385
8.3189	1.0140	1.0156	0.00158
9.3097	1.0100	1.0096	0.00039
10.2163	1.0035	0.9998	0.00371
11.0449	0.9880	0.9839	0.00413
11.8018	0.9630	0.9591	0.00392
12.4929	0.9255	0.9226	0.00287
13.1231	0.8725	0.8723	0.00017
13.6983	0.8075	0.8071	0.00036
14.2221	0.7265	0.7278	0.00129
14.6995	0.6345	0.6362	0.00174
15.1346	0.5345	0.5354	0.00091
15.5311	0.4275	0.4285	0.00099
15.8929	0.3185	0.3184	0.00014
16.2229	0.2085	0.2076	0.00086
16.5241	0.1010	0.0983	0.00271
16.7987	-0.0080	-0.0080	1.1E-05
17.0499	-0.1110	-0.1110	0.00046
17.2793	-0.2090	-0.2080	0.00062
17.4885	-0.3030	-0.3010	0.00205

**Table 6:** Comparison of ACT with other existing methods that extract different parameters of single-diode model intended for Photowatt-PWP201 module.

Methods	*n*	*R*_*s*_ (Ω)	*R*_*p*_ (Ω)	*I*_*λ*_(A)	*I*_*o*_ (μA)	SAE	MAE	RMSE
ACT (proposed)	1.33581	1.2183	762.018	1.03233	3.00257	43.0543E-3	1.65593E-3	2.12633E-3
RF [44]	1.3174002	1.2390187	745.6431	1.0323759	2.5188848	NA	**1.6060E-3**	**2.0465E-3**
PGJAYA [48]	1.3512	1.2013	981.8545	1.0305	3.4818	NA	NA	2.4250E-3
ISCE [37]	1.351189	1.201271	981.98228038	1.0305143	3.48226304	41.78790E-3	NA	2.425075E-3
EHA-NMS [51]	1.351189	1.201271	981.98222618	1.0305143	3.48226292	41.78790E-3	NA	2.425075E-3
R_cr_-IJADE [27]	1.351189	1.201271	981.98216000	1.0305143	3.48226290	NA	NA	2.425075E-3
FPA [43]	1.33698	1.217583	811.3721	1.032091	3.047538	**15.971E-3**	NA	2.054673E-3
MPCOA [63]	1.3474016	1.20295	849.6927	1.03188	3.37370	21.510E-3	2.61E-3	2.425E-3
Newton [23]	1.345833	1.2057	555.5556	1.0318	3.2875	NA	8.32E-3	78.05E-2
PS [64]	1.341358	1.2053	714.2857	1.0313	3.1756	NA	5.3E-3	11.8E-2
IP [13]	1.3106	1.2744	715.824	1.0333	2.3326	NA	1.76E-3	NA
GA [64]	1.34961	1.1968	555.556	1.0441	3.436	15.3479E-2	8.88E-3	NA

Despite the simplicity of implementation and low computational cost the achieved tiny values of simulation errors clearly demonstrated the excellent performance of ACT compared to other reported approaches (Table 6). Moreover, the proposed technique did not require any manual initialization of parameters, while some optimization problems need to be continuously perturbed through parameters variation. This manual initialization results in increased computational cost and often leads to trap the extraction process in local minima. It was affirmed that the proposed ACT is truly competitive to be implemented as a simple and efficient tool for the identification of solar cells and modules parameters operate under diverse environmental conditions.

**Fig 4:**
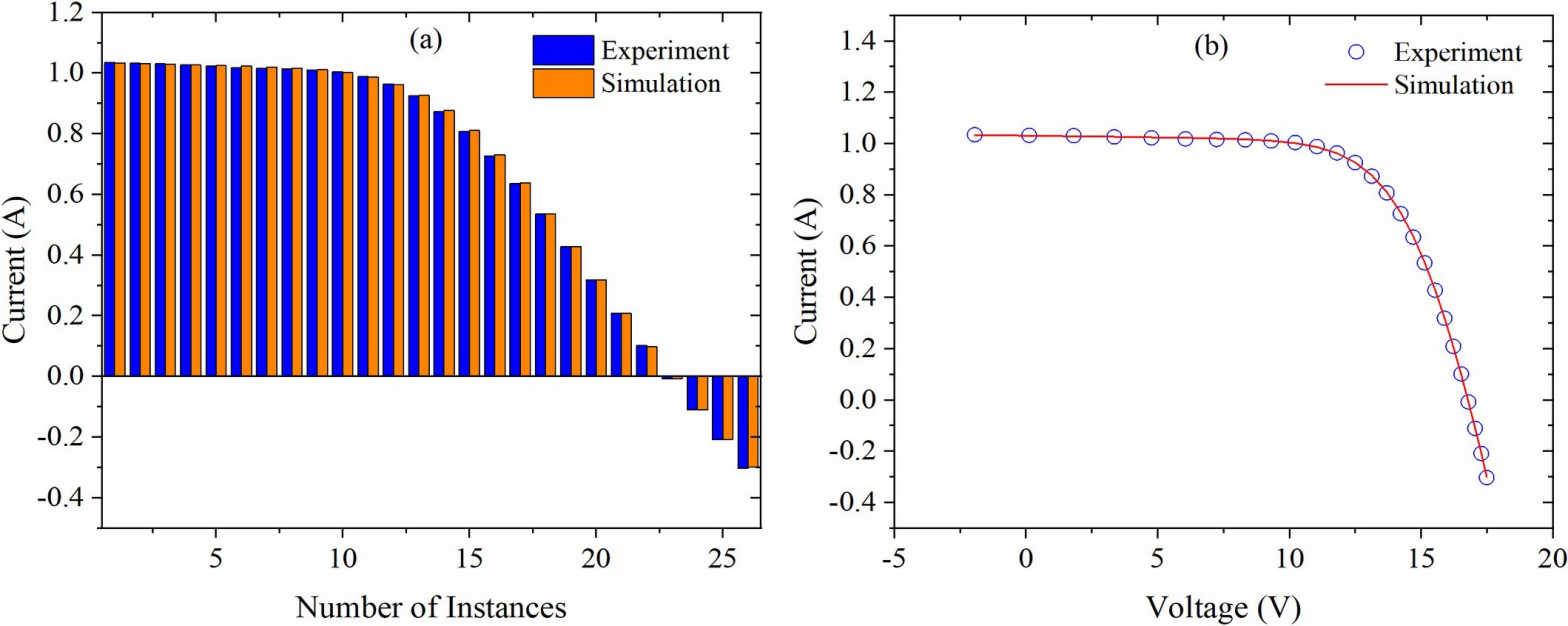
Simulated and experimental currents for single-diode model of Photowatt-PWP201 solar module (a) and their *I-V* curves (b).

Fig 4 shows the ACT based data fitting of Photowatt-PWP201 solar module obtained. The calculated currents are shown to tally very well with the measured ones in both high and low voltage regions, where a trivial deviation around the maximum power point was observed by visual inspection. Such departure could be due to the lacking of extrapolated method applied to identify the values of *V*_*m*_ and *I*_*m*_. The ACT simulation based extracted parameters achieved a lowest RMSE of 2.12633E-3 for Photowatt-PWP201 solar module that too without requiring any manual initialization of parameters. Conversely, some of the existing optimization problems need continuous perturbation through parameters variation, which in turn increases the computational cost and leads to an entrapment in the local optimum.

A mono-crystalline Leibold Solar Module (LSM 20) comprising 20 series cells operated at 24°C under outdoor sunlight intensity of 360 W/m^2^ was tested for its parameters extraction. The measured *I-V* data was given in csv format as the S4 Dataset and recorded manually using digital multimeters. Meanwhile, a potentiometer was utilized as a variable load across the module terminals. The simulated results are listed in Table 7. The extracted parameters were discerned to be *n* = 1.26881, *R*_*s*_ = 6.39445 Ω, *R*_*p*_ = 1973.35 Ω, *I*_*λ*_ = 0.15449 A and *I*_*o*_ = 2.50879 nA. It was asserted that the ACT could effectively model the *I-V* behaviour of the module with a RMSE of 8.3839E-4 and extracted the solar cells parameters in a superior way compared to the one used for the PV modules. It was asserted ACT could outperform the other computational and heuristic algorithms regarding the extraction of single-diode parameters.

**Table 7:** ACT simulation-based results for single-diode model of Leibold Solar Module (LSM 20) operated at 24°C under outdoor sunlight intensity of 360 W/m^2^.

Voltage (V)	Measured Current (A)	Calculated Current (A)	Absolute Error (AE)
0.00	0.154	0.1540	8.8E-06
0.37	0.154	0.1538	0.00020
0.67	0.154	0.1537	0.00035
0.99	0.154	0.1535	0.00051
1.31	0.153	0.1533	0.00033
1.67	0.153	0.1531	0.00015
3.10	0.152	0.1524	0.00042
4.60	0.152	0.1517	0.00035
6.00	0.151	0.1508	0.00015
7.45	0.150	0.1492	0.00081
8.55	0.144	0.1443	0.00031
9.30	0.134	0.1339	6E-05
9.75	0.123	0.1218	0.00117
10.04	0.112	0.1108	0.00125
10.26	0.102	0.1004	0.00157
10.99	0.055	0.0541	0.00088
11.20	0.037	0.0376	0.00059
11.31	0.0281	0.0284	0.00032
11.37	0.0227	0.0233	0.00058
11.41	0.0190	0.0198	0.00080
11.44	0.0160	0.0172	0.00116
11.46	0.0142	0.0154	0.00119
11.48	0.0127	0.0136	0.00091
11.50	0.0120	0.0118	0.00018
11.55	0.0060	0.0073	0.00130
11.58	0.0030	0.0046	0.00156
11.60	0.0020	0.0027	0.00071
11.61	0.0010	0.0018	0.00079
11.62	0.0010	0.0009	0.00014
11.63	0.0006	6.4E-5	0.00066
11.632	0.0003	-2E-04	0.00055
11.633	0.0002	-3E-04	0.00054
11.636	0.0001	-6E-04	0.00072
11.640	0.0001	-1E-03	0.00109
11.650	0.0000	-0.0020	0.00193
SAE	24.2491E-3		
MAE	6.92832E-4		
RMSE	8.3839E-4		

A four series cells-based polycrystalline PV module (STE 4/100) purchased from Leybold GmbH was tested. Experimental *I-V* data of this cell was measured at 22°C under indoor light intensity of 900 W/m^2^. The results for measured *I-V* and simulated *I-V* dataset obtained by the ACT are enlisted in Table 8. Yet again, the proposed ACT could successfully model the *I-V* behaviour of the module with a RMSE of 3.33925E-4. The extracted parameters of this solar module were discerned to be *n* = 1.0304, *R*_*s*_ = 2.5568 Ω, *R*_*sh*_ = 2184.82 Ω, *I*_*λ*_ = 0.02646 A and *I*_*o*_ = 0.129814 nA.

**Table 8:** ACT simulation-based results for single-diode model of Leybold Solar Module (STE 4/100) at 22°C under indoor light intensity of 900 W/m^2^.

Voltage (V)	Experimental Current (A)	Calculated Current (A)	Absolute Error (AE)
0.00	0.0264	0.0264	2.7E-05
0.10	0.0264	0.0264	1.9E-05
0.30	0.0263	0.0263	1E-05
0.50	0.0262	0.0262	1.5E-06
0.70	0.0261	0.0261	6.9E-06
0.90	0.0260	0.0260	1.4E-05
1.00	0.0259	0.0260	6.7E-05
1.10	0.0258	0.0259	0.00012
1.20	0.0257	0.0259	0.00016
1.30	0.0256	0.0258	0.00017
1.40	0.0253	0.0256	0.00033
1.50	0.0252	0.0253	0.00015
1.60	0.0247	0.0247	1.1E-05
1.70	0.0232	0.0231	6.6E-05
1.80	0.0189	0.0196	0.00071
1.90	0.0121	0.0125	0.00038
1.95	0.0081	0.0071	0.00103
2.00	0.0000	0.0003	0.00029
SAE	3.56448E-3		
MAE	1.98027E-4		
RMSE	3.33925E-4		

## Conclusions

A new and simple computational approach based on approximation and correction technique (ACT) was proposed to determine PV cells and modules parameters precisely. Furthermore, a novel user-friendly software application was developed to extract these parameters. The proposed method was tested and validated on five different solar cells and modules under varied temperatures and irradiations. *I-V* simulation of R.T.C. France and PVM 752 GaAs solar cell revealed the lowest RMSE value of 7.78937E-4 and 2.10497E-4, respectively. The Photowatt-PWP201, LEYBOLD 664 431 and STE 4/100 solar modules exhibited the respective RMSE value of 2.12633E-3, 8.3839E-4 and 3.33925E-4. The devised ACT model disclosed excellent performance when tested on various solar modules operated with varied conditions. In addition to implementation simplicity, the method outperformed several previously reported computational and heuristic algorithms used to extract the parameters of single-diode model for solar cells and solar modules under varying environmental conditions.

## Supporting information

S1 Dataset Measured *I-V* data for France solar cell operated at 33°C and 1000 W/m^2^. (CSV)

S2 Dataset Measured *I-V* data for PVM 752 GaAs thin film solar cell operated at 25°C and 1000 W/m^2^. (CSV)

S3 Dataset Measured *I-V* data for Photowatt-PWP201 module operated at 45°C and 1000 W/m^2^. (CSV)

S4 Dataset Measured *I-V* data for Leibold solar module (LSM 20) operated at 24°C under outdoor sunlight intensity of 360 W/m^2^. (CSV)

S5 Dataset Measured *I-V* data for Leibold solar module (STE 4/100) operated at 22°C under indoor light intensity of 900 W/m^2^. (CSV)

S1 File Software application for rapid determination of the PV cells and modules parameters based on approximation and correction technique (ACT). (EXE)
